# Malaria and Soil-Transmitted Helminth Co-Infection and Its Association With Anemia Among Pregnant Women Visiting Mizan–Tepi University Teaching Hospital, Southwest Ethiopia

**DOI:** 10.1155/cjid/3997614

**Published:** 2025-11-25

**Authors:** Tadesse Duguma, Samuel Assefa, Tarekegn Tesfaye, Bezuayehu Alemayehu

**Affiliations:** ^1^Department of Medical Laboratory Science, College of Medicine and Health Sciences, Mizan-Tepi University, Mizan-Aman, Ethiopia; ^2^Department of Pharmacology and Toxicology, School of Pharmacy, College of Medicine and Health Sciences, Mizan-Tepi University, Mizan-Aman, Ethiopia; ^3^Department of Environmental Health, College of Medicine and Health Sciences, Mizan-Tepi University, Mizan-Aman, Ethiopia

**Keywords:** anemia, co-infection, malaria, pregnancy, southwest Ethiopia, STHs

## Abstract

**Background:**

Malaria and soil-transmitted helminth (STH) co-infection have serious public health implications, especially in the sub-Saharan African region, affecting pregnant women. These infections during pregnancy may lead to anemia, which could cause maternal and perinatal complications.

**Objective:**

To assess the prevalence of malaria and STH co-infection and its association with anemia among pregnant women visiting Mizan–Tepi University Teaching Hospital (MTUTH) from September 1 to December 30, 2024.

**Methods:**

An institution-based cross-sectional study was conducted from September 1 to December 30, 2024, among pregnant women attending antenatal care at MTUTH. Blood and stool samples were collected from each pregnant woman using a systematic random sampling method. Sociodemographic and clinical data were collected using a prestructured questionnaire. Bivariate and multivariable logistic regression analyses were used to determine the association between malaria–STH co-infections and anemia. Data entry and analysis were done using Epi-data Version 4.6 and SPSS Version 27.0. A *p* value ≤ 0.05 was considered statistically significant.

**Result:**

The following prevalences were recorded: malaria (24.4%, 81/332), STHs (33.7%, 112/332), and their co-infection (12.7%, 42/332). The overall prevalence of anemia was 57.5%, while it was 18.4%, 26.2%, and 9.6% for pregnant women infected with malaria, STH, and co-infection, respectively. Handwashing practice before meals and residence (rural) showed a significant association with malaria–STH co-infection with (AOR = 12.748, *p* = 0.010) and (AOR = 2.459, *p* = 0.001), respectively. Washing vegetables and fruits before eating has been shown to have an association with malaria–STH co-infection (AOR = 12.420, *p* = 0.017) and (AOR = 0.240, *p* = 0.004), respectively.

**Conclusion:**

The prevalence of malaria, STHs, and their co-infections was high, contributing to the high prevalence of anemia among the study participants. Malaria and STH infection showed a strong statistical association with anemia.

## 1. Introduction

The most common parasitic infections in underdeveloped nations, particularly in sub-Saharan Africa (SSA), are malaria and soil-transmitted helminth (STH) infections, which afflict pregnant women and children under five [1]. The tropical and subtropical regions with high temperatures, stagnant waters, heavy rainfall, and inadequate sanitation all contribute to the parasite's ability to develop and procreate [2]. Malaria and STH infections are linked to anemia, which can result in health complications for both the mother and the fetus [3]. According to the pooled estimates of 253 studies globally, the overall prevalence of malaria in pregnant women was recorded to be 18.95%, with the highest proportion observed in Africa, approximating 21.50% [4]. The 2021 World Health Organization (WHO) report on the global prevalence of anemia in pregnant women indicated that the prevalence of anemia during pregnancy is around 41.8% worldwide, with the highest rates observed in low- and middle-income countries, with 52.5% of pregnant women in Southeast Asia and 19%–34% of pregnant women found to be anemic [5]. Pregnancy-related anemia is characterized by a hemoglobin (Hb) level below 11 g/dL [6]. Throughout pregnancy, the body's Hb level fluctuates; it is low in the first and second trimesters and rises in the third [7, 8].

Anemia during pregnancy is a public health problem, especially in developing countries, which is associated with adverse outcomes for both the mother and child [9]. The prevalence of anemia of ≥ 40% in a population is classified as a severe public health problem [10]. There is a greater risk of anemia during pregnancy due to an increased iron requirement, physiological demand, and loss of blood due to postpartum hemorrhage and infections [11, 12]. In developing countries, anemia during pregnancy is caused by various factors, which include micronutrient deficiencies of iron, folate, and vitamins A and B12; parasitic infections such as malaria and STHs, particularly hookworm; or chronic infections like TB and HIV [13, 14].

The parasitic disease malaria is typically spread by female *Plasmodium*-infected mosquitoes. Any one of the five *Plasmodium* species can infect a human. Most deaths from severe malaria are caused by *Plasmodium falciparum*, whereas moderate malaria is caused by *Plasmodium vivax*, *Plasmodium ovale*, *Plasmodium knowlesi*, and *Plasmodium malariae* [15, 16]. Several studies conducted in Ghana, Nigeria, and Ethiopia noted most malaria infections among pregnant women were caused by *P. falciparum* [17–19]. Research done in Nairobi, Kenya, Nigeria, and East Africa showed that pregnant women with a malaria diagnosis did not use ITNs, which is evidenced by the high burden of both malaria and STH infections in these areas [20–22].

Infections with STHs are a global public health concern in the tropics and subtropics, particularly in many poor countries. In regions with inadequate sanitation, personal hygiene, and a lack of a safe water source, STH infections are prevalent [23]. Pregnant women are primarily affected by three types of STHs: hookworms, whipworms (*Trichuris trichiura*), and roundworms (*Ascaris lumbricoides*) [24]. In order to decrease transmission and reinfection by promoting healthy habits, the WHO advised proper sanitation and health education [25]. The goal of preventive chemotherapy during pregnancy is to lessen the severity of the infection and shield expectant mothers from the morbidity linked to STH infections. Albendazole (400 mg) and mebendazole (500 mg) are the two anthelmintics that are advised for the treatment of STH infections during pregnancy since they are reasonably priced, accessible in medical facilities, efficient, simple to use, and have fewer adverse effects [26]. Wearing shoes, wearing protective clothes, and using clean water all help to lower the risk of contracting schistosomiasis and hookworm [27].

The STHs remain a massive hidden burden, affecting over 1.5 billion people worldwide, especially in low-resource tropical and subtropical regions, particularly in SSA, the Americas, China, and East Asia, affecting vulnerable groups like preschool and school-age children (due to exposure and hygiene practices) and women of reproductive age, especially during pregnancy [28].

Malaria causes ∼249 million cases and over 600,000 deaths annually, with the heaviest toll in SSA. Both conditions overlap geographically and contribute heavily to anemia and poor maternal-child health outcomes [29].

Despite pregnant women obtaining preventive and treatment services, the prevalence of anemia at MTUTH has increased (unpublished data sources available at the hospital's ANC). The prevalence of co-infections between malaria and STHs and their relationship to anemia in pregnant women in the area have not been the subject of any published research. Information is required to determine the causes of the high incidence of anemia in the area despite the implementation of preventive and treatment measures, as well as whether malaria and STH co-infections with anemia have an association. This study may provide baseline information for developing community awareness-raising campaigns regarding the prevention and control of malaria and STH infections and their link to anemia.

## 2. Materials and Methods

### 2.1. Study Design, Setting, and Period

An institutional cross-sectional study was conducted among pregnant women who received antenatal care (ANC) services at Mizan–Tepi University Teaching Hospital (MTUTH) between September 1 and December 30, 2024. MTUTH is a university hospital that was founded in 1986. The hospital was previously known by its name under the Derg government, “Mizan–Teferi General Hospital.” It is situated in the town of Mizan–Aman in the southwest region of Ethiopia. Currently it is serving a catchment population of approximately 2–2.75 million people from multiple zones, including Bench Sheko, Kaffa, Sheka, and Majang, with a total of 602 staff and providing a variety of services for approximately 350 clients on a daily basis across the different departments. Mizan–Aman, located in the Bench Sheko Zone of southwest Ethiopia, has a hot climate with a prolonged rainy season lasting 9 months. The town experiences average annual rainfall ranging from 400 to 2000 mm, sometimes leading to flooding due to limited drainage infrastructure. The average annual temperature is around 19.6°C, largely due to deforestation and urban expansion. Mizan–Aman is known for its coffee production, supported by fertile soils and favorable rainfall. It is the capital of the Bench Sheko Zone and is situated 565 km southwest of Ethiopia's capital, Addis Ababa (Wikipedia).

### 2.2. Study Variables

Anemia is the dependent variable, which is influenced by malaria and STH co-infection, while other variables, such as sociodemographic and economic characteristics, include age, marital status, residence, education level, employment status, and occupational status.

Obstetric characteristics include trimester, gravidity status, and the number of ANC visits.

Behavioral habits include handwashing before meals and after visiting the toilet, the habit of biting nails, eating raw and unwashed vegetables, eating soil/rocks, water drinking sources, water preservation methods, use of ITNs, and malaria chemoprophylaxis, while environmental factors include soil contact and sanitary facilities. Clearing of bushes and draining of stagnant water.

### 2.3. Study Population

All pregnant women attending ANC at MTUTH were included in our study.

### 2.4. Inclusion Criteria

Pregnant women who were attending antenatal clinic during the study period and who were willing to participate and provide informed consent.

### 2.5. Exclusion Criteria

Pregnant women who were on hematinic, antimalarial, and anthelmintic medications and who did not give their consent for participation. Moreover, pregnant women with pre-existing hematological disorders, chronic illnesses, recent blood transfusions, or those unwilling or unable to provide informed consent were excluded.

### 2.6. Sample Size and Sampling Technique

#### 2.6.1. Sample Size Determination

The sample size was calculated using the single population proportion formula as follows by taking the previous study conducted on the prevalence of soil-transmitted malaria co-infection among pregnant women in the Gilgel Gibe dam area, southwest Ethiopia (*p* = 7.7%) [29]:

(1) $$\begin{array}{c} n=z^{2}p\frac{\left(1-P\right)}{d^{2}}, \end{array}$$

where *n* = minimum sample size required, *Z* = *Z* scores corresponding to the level of confidence (95% = 1.96), *P* = the proportion in the target population estimated to have the characteristic being measured, *d* = margin error = 3%, and *q* = 0.923.

(2) $$\begin{array}{c} n=\frac{{\left(1.96\right)}^{2}*0.077*\left(1-0.077\right)}{{\left(0.03\right)}^{2}}=303.4, \end{array}$$

where *n* = 303.4≈304. Adding a 10% nonresponse rate [30], 304 + 30.4 = 334.4≈335.

#### 2.6.2. Sampling Technique

The systematic random sampling technique was employed to select pregnant women. Stool and blood samples were collected from each study participant to investigate the prevalence of malaria, helminth infections, co-infections, and anemia. Checking the ANC attendants' logbook, the client flow for the past two months was assessed, and it was found that 896 (*N*) pregnant women were attended to at the ANC clinic; accordingly, the sampling interval was calculated as (*K* = *N*/*n*), (896/335 = 2.67≈3). After random selection of the initial study participant through a lottery method, we proceeded to enroll every third pregnant woman (*k* = 3) who visited the hospital until we reached the desired sample size. The collected blood samples were also used to check the Hb level and investigate the maternal anemia.

### 2.7. Data Collection and Laboratory Procedure

#### 2.7.1. Questionnaires on Sociodemographic and Clinical Data Collection Procedure

A well-structured and designed pretested questionnaire, which contained questions related to sociodemographic and economic characteristics, behavioral habits, and environmental conditions, was used to collect information about pregnant women. The questionnaire was comprised of closed and open-ended questions.

The investigator and data collectors read the question from the questionnaire to the participant, allowed her to respond, and then recorded the answers.

#### 2.7.2. Specimen Collection and Laboratory Methods

All consenting pregnant women were provided with labeled screw-capped stool containers, which will be labeled with the code number of each participant. Clear instructions were given on how to collect the stool and transfer about 5 g of fresh stool sample into a container.

#### 2.7.3. Malaria Parasite Examination and Parasite Identification

About 5 mL of blood was drawn into an EDTA anticoagulant tube aseptically using a sterile syringe and needle from each study participant. One drop of the blood was put on a sterile glass slide to make a thick and thin film of blood for the malaria parasite identification, and it was then allowed to air dry horizontally. The thin blood film was prepared and fixed with 70% methanol for 30 s and air-dried and examined microscopically to identify *Plasmodium* species. Accordingly, a malaria parasite (*P. falciparum*) with small, delicate rings and appliqué (peripheral) forms in thin blood films, often with multiple infections per normal-sized red blood cell with crescent- or banana-shaped gametocytes, may have coarse Maurer's dots, while *P. vivax* infects distorted red blood cells with a larger ring form and thicker cytoplasm and large amoeboid trophozoites with fine golden pigment have large, round, or oval *P. vivax* gametocytes fill enlarged RBCs and have fine, regular Schüffner's dots. Both thin and thick films were stained using 10% Giemsa for 10 min and examined using an oil immersion objective (100^x^).

#### 2.7.4. Estimation of Hb Levels

The maternal Hb level was measured using a CBC machine (Mindray Medical International Limited, China) by putting 1 mL of blood in an EDTA test tube. The result was immediately displayed on screen, and the Hb concentration of each subject was read within 1 minute. Control blood samples with known values of low Hb (Hb < 11.0 g/dL) and high Hb (Hb 18 g/dL) were run daily to validate the accuracy and reliability of the CBC machine results.

### 2.8. Quality Control

Training was given to the data collectors and supervisors to ensure the quality of the data used in the study, and the principal investigator and supervisors conducted routine oversight and follow-up. Additionally, the data were regularly checked for consistency and completeness every day. A clinical and sociodemographic questionnaire was prepared in English, translated into Amharic and Benchigna, and then back to English to guarantee correctness and relevance. The reagents and kits were evaluated using known positive and negative controls in accordance with the standard operating procedure (SOP) for each test method. Data confidentiality was maintained, and a code was used to reveal the outcome. For every outcome, a cross-check was performed. To maintain quality, the Giemsa was filtered and 10% buffered (pH 7.2) and prepared for use every 24 h.

### 2.9. Data Analysis and Interpretation

Epi-data Version 4.6 was used to code, enter, and verify the data before they were exported into SPSS Version 27.0 for analysis. The prevalence and severity of anemia, parasitic infections and their coinfections, behavioral and environmental characteristics, and sociodemographic and obstetric characteristics were all examined using descriptive and inferential statistics. The relationship between malaria, STH infection, their co-infection, and anemia was assessed using bivariate and multivariable logistic regression. Statistical significance was defined as a *p* value of less than 0.05.

### 2.10. Ethical Approval

Ethical clearance with approval number CMHS/00992/24 was obtained from the College of Medicine and Health Sciences. Participants of the study were informed about the purpose of the investigation and provided written informed consent. Participants' identifiers were not written on the questionnaire in order to maintain the confidentiality of their information, and they were interviewed separately to protect personal privacy. Generally, the study was conducted in accordance with the Declaration of Helsinki (1964).

### 2.11. Operational Definition of Terms

Malaria: It is a parasitic infection commonly transmitted by infected female *Anopheles* mosquitoes, which belong to the *Plasmodium* type.

STHs: These are soil-transmitted worms that can infect the gastrointestinal tract of humans.

Co-infection: Simultaneous infection of a host organism by two or more pathogens.

Anemia: Hb level of less than 11 g/dL (Hct < 30%) [6].

Mild anemia: categorized as an Hb level between 9 and 12.0 g/dL.

Moderate Anemia: defined as an Hb level between 7 and 8.9 g/dL.

Severe Anemia: categorized as an Hb level less than 7 g/dL.

Prevalence: It is the proportion of persons in a population who manifest disease at a specific point in time or over a specific period.

Pregnant Women: A woman having a developing embryo, fetus, or unborn offspring within the body.

## 3. Results

### 3.1. Socioeconomic and Demographic Characteristics

Data were collected from 332 pregnant women, and a response rate of 99.1% was recorded. The mean and standard deviation of the age of the study participants was 28.48 (SD = 6.470), with more than one-third aged between 25 and 29 years. More than half of the study participants were from rural areas, while 47.6% were urban residents. The majority of pregnant women were married, and more than a quarter were illiterate. One hundred thirteen (34.0%) pregnant women were housewives. One hundred and ninety (57.2%) pregnant women had at least attended the antenatal clinic once (Table 1).

### 3.2. Health-Seeking Behavior of the Study Participants

Nineteen (5.7%) of the ANC attendants never visit the institution for the service, while 25 (7.5%) of them do not seek health services when they feel febrile, and nearly three-fourths of the pregnant women experience abdominal discomfort and seek health services for it. Only slightly below half (48.5%) of the study participants reported that they were treated for anemia during their gestational period (Table 2).

### 3.3. Prevalence of Anemia During Pregnancy

**Figure 1:**
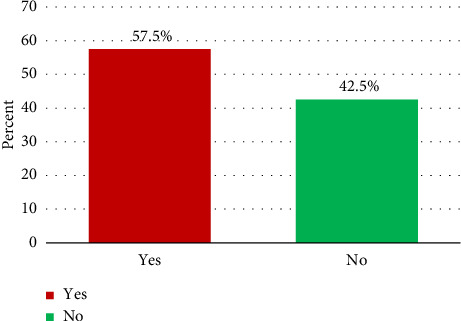
Anemia during pregnancy among women attending the ANC clinic at MTUTH, Southwest Ethiopia.

Anemia was defined as an Hb level value < 11.0 g/dL [6]. One hundred ninety-one (57.5%) pregnant women had anemia, of whom 111 (33.4%), 69 (20.8%), and 11 (3.3%) represented mild, moderate, and severe anemia, respectively, while one hundred forty-one (42.5%) pregnant women had no anemia (Figure 1).

### 3.4. Clinical Characteristics of the Pregnant Women

Among the pregnant women who had malaria infection, 61 had anemia, while 20 of them had no anemia, while the remaining participants were not infected. Among the pregnant women who had STH infection, more than three-fourths were found to have anemia, while more than one-fifth had no anemia. Nearly one-fourth of the pregnant women had malaria infections, of which forty-five (13.6%) were caused by *P. falciparum*, followed by *P. vivax* and mixed infections (Table 3).

### 3.5. Behavioral Measures Used to Prevent Malaria and STH Infection

Nearly half of the pregnant women used ITN, and 20.2% of non-users reported that they have no access to ITN. More than half of the study participants reported their houses were not sprayed. More than two-thirds of the study participants reported that they regularly wash their hands before meals, and about two-thirds of them reported that they wash their hands after visiting the toilet. More than three-fourths of the pregnant women reported they washed vegetables before eating. About 229 (69.0%) of pregnant women reported that they did not use any water preservation methods. All of the study participants reported that they have a latrine facility in their residence (Table 4).

### 3.6. Prevalence of Malaria, STH Infection, Co-Infection, and Anemia Among Pregnant Women

**Figure 2:**
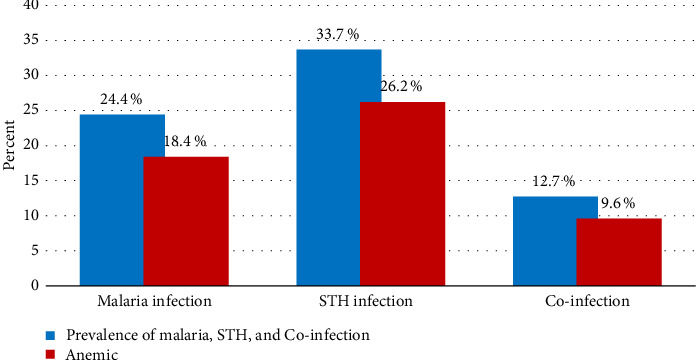
Prevalence of anemia among pregnant women infected with malaria, STH, and their co-infection attending the ANC clinic at MTUTH, Southwest Ethiopia.

About 81 (24.4%) pregnant women were found to have malaria infection, while STH infection was found in 112 (33.7%) of the study participants, of which thirty-five (10.5%) of the study participants were infected with *A. lumbricoides*, twenty-four (7.2%) with *T. trichiura*, twenty (6.0%) with hookworm, six (1.8%) with *Hymenolepis* species, twelve (3.6%) with *Taenia species,* and fifteen (4.5%) with *Schistosoma mansoni*. But none of the pregnant women had multiple STH infections. Moreover, co-infection was recorded in 42 (12.7%) pregnant women who participated in the study. Sixty-one (18.4%) pregnant women were found to have anemia of participants infected with malaria, while 87 (26.2%) of them were found anemic from STH-infected individuals. Thirty-two (9.6%) of women had anemia from groups infected by both malaria and STHs. Additionally, anemia was identified in study participants who were not infected by malaria, STH, or both, with a prevalence of 130 (51.8%), 104 (47.3%), and 159 (54.8%), respectively (Figure 2).

### 3.7. Sociodemographic Factors Associated With Malaria and STH Co-Infection

Sixteen (4.8%) of the pregnant women aged 25–29 years had malaria and STH co-infection. Twenty-nine (8.7%) pregnant women who had malaria and STH co-infection were from rural areas. Of the sociodemographic factors, age (20–24 years) and residence (rural) showed statistically significant association with malaria–STH co-infection (Table 5).

### 3.8. Factors Associated With Malaria and STH Co-Infection

Handwashing before meals, washing vegetables and fruits before eating, and the habit of fingernail biting showed a statistically strong association with malaria–STH co-infection. A high magnitude of malaria–STH infection was observed among ITN and IRS non-users (see Table 6).

### 3.9. Malaria, STH, and Their Co-Infection, With Respect to Severity of Anemia

Among the pregnant women who had malaria, twenty-eight (8.4%) pregnant women had mild anemia, 22 (6.6%) pregnant women had moderate anemia, 11 (3.3%) pregnant women had severe anemia, and 101 (30.4%) who had no malaria did not have anemia. Of those who had STHs, 51 (15.4%) pregnant women had mild anemia, 28 (8.4%) pregnant women had moderate anemia, and eight (2.4%) had severe anemia. Among the pregnant women who had malaria and STH co-infection, 10 (3.0%) pregnant women had mild anemia, 13 (3.9%) pregnant women had moderate anemia, and 9 (2.7%) pregnant women had severe anemia. This study revealed that malaria and STH infection were found to have a significant association with anemia prevalence, but an association was not observed in their co-infection (Table 7).

## 4. Discussion

The co-occurrence of malaria and STH has a significant impact on pregnant women's health, and anemia is one of the most dangerous problems that can have a major negative impact on the mother and fetus. A total of 332 pregnant women participated, and a 99.1% response rate was recorded. According to the current study, almost one-third of the participants had STH infections, and over a quarter had malaria parasite infections. The prevalence of anemia in pregnant women remains a significant global health problem, with a reported rate of 57.5% in a recent study. This finding is aligned with a systematic review that reveals high anemia rates, approximately 37%–75% in different populations [31]. For example, a national survey in China reported a similar prevalence of 46.5% [32]. Additional research stressed that 41% of pregnant women in Bangladesh experienced anemia [33], while Ethiopia reported a 32% prevalence [34]. In Egypt, a study found that 61% of pregnant women suffered from iron deficiency anemia, suggesting that socioeconomic and nutritional factors contribute significantly to these rates [35]. These findings underline the urgent need for specific interventions to mitigate anemia among pregnant populations worldwide.

Additionally, another study from Nairobi, Kenya, reported the prevalence of anemia during pregnancy as 40.7% [36], which is lower compared to the current study, but two more study findings from Ghana and coastal Kenya revealed a higher prevalence of 66.4% and 71.0% [37, 38], respectively. Anemia during pregnancy remains a problem in Ethiopia and our study area. Maternal anemia during pregnancy is mostly caused by malaria, particularly in regions where the disease is endemic [39].

In comparison to studies conducted in Nigeria, western Kenya, and Ghana, which reported prevalences of malaria infection during pregnancy of 26.5%, 21.6%, and 16.5%, respectively. This study found the prevalence of malaria infection among pregnant women to be 24.4%, which is somewhat higher [38–41]. The availability and variation in the usage of malaria control measures (ITN, IRS, and insect repellents), which were less than 50% in terms of use, were low as demonstrated in our study. *P. falciparum* was the most common species among research participants, contributing to 12.6% of the malaria infections, followed by *P. vivax,* which infected 7.2% of the pregnant women. This concurs with findings of studies in different parts of Ethiopia, Ghana, and Nigeria, which noted most of the malaria infections among pregnant women were caused by *P. falciparum* [23, 39, 41, 42].

Effective health interventions and continuous research are essential to alleviate the complications associated with malaria during pregnancy.

The WHO advises using ITNs during pregnancy as a means of preventing malaria infection because sleeping under an ITN reduces human interaction with mosquitoes, which lowers the risk of contracting malaria and the related mortality for both the mother and the fetus [43]. In line with other research from Nairobi in Kenya, Nigeria, and Cameroon, this study found that the majority of pregnant women did not use mosquito repellents as a malaria preventive method, and more than half of study participants did not utilize ITN and IRS [37, 42, 44]. The study participants mentioned the different reasons why they did not use the ITN, such as lack of interest, because it was torn, or having no access. According to research conducted in Nigeria, pregnant women who did not use bed nets reported experiencing excessive sweating at night, which was caused by inadequate ventilation [23]. This implies that use of ITN is an effective means of controlling malaria infection among pregnant women.

Infection with STHs is attributed to contamination of soil and water with cysts, ova, and larvae of the parasites. The ova of STHs are transmitted by ingesting food, water, or soil/rocks and through contaminated fingers. The prevalence of STH infection in this study was 33.7%, which is lower than findings of studies in other parts of Ethiopia (51%) but slightly higher than findings of studies in Nigeria (12.0%) and Western Kenya (24.7%) while consistent with findings from Nepal (33.7%) [17, 38, 45, 46]. The difference in prevalence of STHs can be attributed to the fact that different communities are exposed to different parasitic infections. Among pregnant women who had STH infections, *A. lumbricoides* was the most predominant helminth. This agrees with findings from studies in western Kenya, Ghana, and Nigeria, which noted *A. lumbricoides* was the most predominant parasite among the pregnant women [36, 38, 45]. Results from research conducted in Kenya's coastal and northern Rift Valleys as well as other regions of Ethiopia showed that pregnant women living in rural areas had a higher risk of contracting the disease because of inadequate waste management, low socioeconomic status, and unsanitary environments, which is in line with the finding of our study with 2.033 times more vulnerability in rural residents compared to their counterparts [46–49]. Pregnant women should be encouraged to continue using the latrine to dispose of human waste and to wash their hands, important measures to prevent STH infection.

Co-infection with STHs and malaria showed an association with anemia, which may have considerable health effects leading to more severe clinical symptoms and pathology. The finding from the current study is lower than findings from Nigeria, Niger, Ethiopia, and Ghana, which were 43.1%, 28%, 19.5%, and 16.6% [14, 50–52], respectively. The current finding is higher compared to studies from Nigeria, western Kenya, and Ethiopia, which were 5.5%, 6.8%, and 7.7% [38, 41, 53], respectively. The higher prevalence can be attributed to factors such as geographical variations, climatic conditions, and risks associated with transmission of the infection. The co-infection rate of 12.7% in our study seems relatively low compared with other geographical areas. Such disparity could be due to variance in local transmission patterns, socioeconomic factors, and the impact of public health measures. But at this reduced prevalence, co-infections may still play a role in adverse pregnancy outcomes such as anemia, low birth weight, and prematurity. Regular monitoring and targeted interventions are needed to minimize the impact of these co-infections on maternal and fetal health. This study revealed that being a rural resident has a 2.459 times higher risk of getting a co-infection by malaria and STH compared to their counterparts.

Results of an Ethiopian investigation showed that the epidemiology of clinical malaria was impacted by helminth infection [54]. This study indicated *P. falciparum* and *A. lumbricoides* were the most prevalent co-infections among expectant mothers. This is consistent with research conducted in Nigeria that found *P. falciparum* and *A. lumbricoides* to be the most common co-infections among pregnant women [14]. Among the pregnant women who were co-infected, 9.6% were anemic, of which 3.3% were severe anemia cases. That agrees with the findings of a study conducted in western Kenya, which noted coinfection of STHs and *P. falciparum* to have an association with maternal anemia [49, 55]. The multivariable logistic regression analysis showed that handwashing before meals was found to have a strong association with STH infection, which agrees with the findings from studies in Ethiopia and the Coastal and Northern Rift Valley in Kenya, which noted pregnant women who washed their hands thoroughly after visiting the toilet were not infected with STHs [46, 48, 49].

Pregnant women who had malaria infection were 2.9 times more likely to have severe anemia than those who had no malaria. Since the area is a malaria-endemic region, pregnant women have a higher risk of being infected with the malaria parasite [56]. Pregnant women should be advised to adhere to measures in the prevention of malaria and STH infection, which can lead to mild, moderate, and severe anemia. Infection with STHs, particularly hookworm (6.0%) and *A. lumbricoides* (10.5%), two of the prevalent STHs in this study, contributed to the anemia during pregnancy, which may affect maternal and fetal health [57]. According to WHO, STH infections during pregnancy lead to increased nutrient malabsorption rates, competing for vitamin A in the intestines. Infected pregnant women usually have no appetite, hence diminished nutritional intake, which can cause anemia [43]. Co-infection with malaria and STH infections may lead to a more severe form of anemia than infection with malaria or STHs alone, as is evidenced from the current study and agrees with findings from a study in western Kenya, which noted pregnant women who had geohelminths and malaria co-infection were four times more likely to have anemia compared to those who had geohelminths or malaria alone [38]. Pregnant women should be encouraged to attend ANC so that they can receive routine services offered, such as malaria chemoprophylaxis and insecticide-treated nets. They should also be educated on factors associated with malaria and intestinal coinfection and advised to practice personal hygiene and environmental sanitation to prevent the infection.

## 5. Conclusion

The prevalence of anemia was 57.5% among pregnant women at MTUTH, and the mild type of anemia persists mostly in the last two trimesters of pregnancy. The prevalence of malaria, STHs, and their coinfection was also high despite preventive measures that have been put in place by the government to reduce the transmission. Use of mosquito repellents and residence (rural) were found to have an association with malaria infection, while the practice of handwashing before meals and after toilet showed an association with STH infection. Moreover, this study noted that malaria and STH infections have a strong association with anemia among the study participants.

### 5.1. Recommendation

Early diagnosis of anemia by encouraging pregnant women to attend antenatal clinics early, prophylaxis by offering routine iron supplements and treatment, and dietary counseling for anemia in pregnant women should be undertaken to reduce the prevalence of anemia. Pregnant women attending antenatal clinics should be screened for STH infection through routine stool tests and offered appropriate treatment in case of infection. Providing health education on good personal hygiene is important in addition to prompt treatment of pregnant women who have malaria and STH infections to reduce anemia prevalence.

## Figures and Tables

**Table 1:** The sociodemographic and obstetric characteristics of pregnant women attending ANC clinic at MTUTH (*n* = 332).

Characteristics	Category	Frequency (%)
Age (years)	15–19	12 (3.6)
	20–24	64 (19.5)
	25–29	130 (39.2)
	30–34	65 (19.6)
	35–39	36 (10.8)
	≥ 40	25 (7.5)

Residence	Urban	158 (47.6)
	Rural	174 (52.4)

Marital status	Single	5 (1.5)
	Married	324 (97.6)
	Separated	3 (0.9)

Educational status	Illiterate	90 (27.1)
	Primary	133 (40.1)
	Secondary	73 (22.0)
	College and above	36 (10.8)

Occupational status	Housewife	113 (34.0)
	Farmer	133 (40.1)
	Merchant	61 (18.4)
	Employed	25 (7.5)

Family monthly income in Ethiopian birr (ETB)	Low (< 1000)	229 (69.0)
	Middle (1001–5000)	82 (24.7)
	High (> 5000)	21 (6.3)

Family size	< 5 members	98 (29.5)
	≥ 5 members	234 (70.5)

Trimester	First trimester	83 (25.0)
	Second trimester	141 (42.5)
	Third trimester	108 (32.5)

Gravidity status	Primigravida gravidae	144 (43.4)
	Multigravida	188 (56.6)

*Note:* ETB = Ethiopian birr.

**Table 2:** Health-seeking behavior of the study participants (*n* = 332).

Variable (questions)	Category	Frequency (%)
Have you visited a health facility for ANC service before?	Yes	313 (94.3)
	No	19 (5.7)

Number of health facility visits	< 4 times	190 (57.2)
	4 times	97 (29.2)
	> 4 times	26 (7.8)

Seek health services when feeling febrile	Yes	307 (92.5)
	No	25 (7.5)

Seeks health service when feeling abdominal discomfort	Yes	246 (74.1)
	No	86 (25.9)

How you experienced the following in the past	Tiredness	117 (35.2)
	Shortness of breath	91 (27.4)
	Rapid heartbeats	61 (18.4)
	Blood in urine	24 (7.2)
	Dizziness	39 (11.7)

Have you been treated for anemia during pregnancy?	Yes	161 (48.5)
	No	171 (51.5)

Do you receive iron supplements during pregnancy?	Yes	111 (33.4)
	No	221 (66.6)

Previous history of malaria infection during pregnancy?	Yes	49 (14.8)
	No	283 (85.2)

Have you received intermittent preventive therapy for malaria?	Yes	36 (10.8)
	No	296 (89.2)

**Table 3:** Clinical characteristics of the study participants (*n* = 332).

Variables	Category	Anemic (%)	Non-anemic (%)
Malaria infection	Yes	61 (75.3)	20 (24.7)
	No	130 (51.8)	121 (48.2)

STH infection	Yes	87 (77.7)	25 (22.3)
	No	104 (47.3)	116 (52.7)

Malaria–STH co-infection	Yes	32 (76.2)	10 (23.8)
	No	159 (54.8)	131 (45.2)

**Malaria prevalence and species distribution**			
**Variables**	**Category**	**Frequency**	**Percentage**

Malaria parasite found	Yes	81	24.4
	No	251	75.6

*Plasmodium* species identified	*Plasmodium falciparum*	45	13.6
	*Plasmodium vivax*	24	7.2
	Mixed infection (P.f. + P.v.)	12	3.6

**Table 4:** Behavioral measures used to prevent malaria and STH infection by pregnant women attending ANC clinic at MTUTH (*n* = 332).

Behavioral measures	Category	Frequency (%)
Use of ITN	Yes	164 (49.4)
	No	168 (50.6)

Reasons for not using a mosquito net	Did not have ITN	67 (20.2)
	Torn	55 (16.6)
	Not available to buy	46 (13.9)

Use mosquito repellent	Yes	20 (6.0)
	No	312 (94.0)

Reason for not using?	Not available in the area	292 (88.0)
	Did not believe it can prevent mosquito bite	40 (12.0)

Use of indoor residual spray	Yes	138 (41.6)
	No	194 (58.4)

Washing before a meal	Regularly	233 (70.2)
	Sometimes	99 (29.8)

Handwashing after visiting the toilet	Regularly	219 (66.0)
	Sometimes	113 (34.0)

Wash vegetables before eating	Yes	263 (79.2)
	No	69 (20.8)

Wash fruits before eating	Always	170 (51.2)
	Sometimes	127 (38.3)
	Not at all	35 (10.5)

Eating soil/rocks	Yes	33 (9.9)
	No	299 (90.1)

Habit of biting fingernails	Yes	42 (12.7)
	No	290 (87.3)

Practice of the shoe-wearing habit	Yes	258 (77.7)
	No	74 (22.3)

Source of water for drinking	River	25 (7.5)
	Tap water	195 (58.7)
	Well	70 (21.1)
	Stream	42 (12.7)

Use of water preservation methods (chlorinating, filtering, and boiling)	Yes	103 (31.0)
	No	229 (69.0)

Do you have a toilet or latrine around in your houses?		332 (100.0)

**Table 5:** Sociodemographic factors associated with malaria and STH co-infection among pregnant women attending ANC at MTUTH (*n* = 332).

Malaria and STH co-infection				Odds ratio (95% CI)			
Characteristics	Category	Yes *n* (%)	No *n* (%)	COR	*p* value	AOR	*p* value
Age (years)	15–19	3 (0.9)	9 (2.7)	Ref		Ref	
	20–24	9 (2.7)	55 (16.6)	2.037 (0.462–8.990)	0.348	0.298 (0.102–0.872)	0.027^**∗**^
	25–29	16 (4.8)	114 (34.3)	2.375 (0.581–9.704)	0.228	0.756 (0.329–1.735)	0.509
	30–34	6 (1.8)	59 (17.8)	3.278 (0.693–15.494)	0.134	0.928 (0.425–2.027)	0.852
	35–40	5 (1.5)	31 (9.3)	2.067 (0.412–10.363)	0.378	1.306 (0.546–3.123)	0.549
	> 40	3 (0.9)	22 (6.6)	2.444 (0.413–14.471)	0.325	1.001 (0.403–2.488)	0.998

Residence	Urban	15 (4.5)	143 (43.1)	Ref		Ref	
	Rural	27 (8.1)	147 (44.3)	0.448 (0.224–0.897)	0.023	2.459 (1.610–3.754)	0.001^**∗**^

Educational status	Illiterate	10 (3.0)	80 (24.1)	0.727 (0.188–2.812)	0.644	1.361 (0.526–3.521)	0.525
	Primary	20 (6.0)	113 (34.0)	0.514 (0.144–1.836)	0.305	2.004 (0.820–4.898)	0.128
	Secondary	9 (2.7)	64 (19.3)	0.646 (0.164–2.550)	0.533	1.539 (0.588–4.027)	0.380
	College and above	3 (0.9)	33 (9.9)	Ref		Ref	

Occupational status	Housewife	17 (5.1)	96 (28.9)	0.491 (0.106–2.277)	0.364	1.927 (0.659–5.632)	0.231
	Farmer	14 (4.2)	119 (35.8)	0.739 (0.157–3.473)	0.702	1.207 (0.406–3.581)	0.735
	Merchant	9 (2.7)	52 (15.7)	0.502 (0.101–2.511)	0.402	1.849 (0.598–5.720)	0.286
	Employer	2 (0.6)	23 (6.9)	Ref		Ref	

Family average monthly income (birr)	Low (< 1000)	26 (7.8)	203 (61.1)	2.440 (0.825–7.214)	0.107	0.433 (0.167–1.125)	0.086
	Middle (1001–5000)	11 (3.3)	71 (21.4)	2.017 (0.615–6.618)	0.247	0.501 (0.176–1.425)	0.195
	High (> 5000)	5 (1.5)	16 (4.8)	Ref		Ref	

*Note:* Reference category selection: Reference category was based on the burden of disease among the categories (small disease burden was selected as reference category). Ref = Reference category.

Abbreviations: AOR, adjusted odds ratio; CI, confidence interval; COR, crude odds ratio.

^∗^Significant association.

**Table 6:** Factors associated with malaria and STH co-infection among pregnant women attending ANC at MTUTH (*n* = 332).

Malaria and STH co-infection				Odds ratio (95% CI)			
Variables	Category	Yes *n* (%)	No *n* (%)	COR	*p* value	AOR	*p* value
Handwashing before meals?	Regularly	9 (2.7%)	224 (67.5)	Ref		Ref	
	Sometimes	33 (9.9)	66 (19.9)	0.080 (0.037–0.176)	0.001	12.748 (4.379–37.109)	0.010^∗^

Handwashing after visiting the toilet?	Yes	18 (5.4)	201 (60.5)	Ref		Ref	
	No	24 (7.2)	89 (26.8)	0.332 (0.172–0.643)	0.001	1.458 (0.540–3.936)	0.457

Washing vegetables before eating?	Yes	41 (12.3)	222 (66.9)	0.080 (0.011–0.590)	0.013	12.420 (1.557–99.086)	0.017^∗^
	No	1 (0.3)	68 (20.5)	Ref		Ref	

Washing fruits before eating?	Always	14 (4.2)	156 (47.0)	3.857 (1.515–9.822)	0.005	0.240 (0.090–0.642)	0.004^∗^
	Sometimes	19 (5.7)	108 (32.5)	1.96 (0.799–4.846)	0.141	0.497 (0.184–1.340)	0.167
	Not at all	9 (2.7)	26 (7.8)	Ref		Ref	

Practice of the shoe-wearing habit?	Yes	26 (7.8)	232 (69.9)	2.462 (1.240–4.888)	0.010	0.563 (0.261–1.214)	0.143
	No	16 (4.8)	58 (17.5)	Ref		Ref	

Eating soil/rocks	Yes	3 (0.9)	30 (9.0)	Ref		Ref	
	No	39 (11.7)	260 (78.3)	0.667 (0.194–2.289)	0.519		

Habit of biting fingernails	Yes	14 (4.2)	28 (8.4)	Ref		Ref	
	No	28 (8.4)	262 (78.9)	4.679 (2.209–9.911)	0.001	0.174 (0.055–0.549)	0.003^∗^

Source of drinking water	River	5 (1.5)	20 (6.0)	Ref		Ref	
	Tap water	27 (8.1)	168 (50.6)	1.556 (0.538–4.494)	0.414	0.787 (0.118–5.255)	0.805
	Well	4 (1.2)	66 (19.9)	4.125 (1.010–16.841)	0.048	0.254 (0.056–1.154)	0.076
	Stream	6 (1.8)	36 (10.8)	1.500 (0.406–5.541)	0.543	1.403 (0.223–8.826)	0.718

Use of water preservation methods	Yes	17 (5.1)	86 (25.9)	Ref		Ref	
	No	25 (7.5)	204 (61.4)	1.613 (0.829–3.139)	0.159	0.742 (0.290–1.899)	0.534

Use of ITN	Yes	13 (3.9)	151 (45.5)	Ref		Ref	
	No	29 (8.7)	139 (41.9)	0.413 (0.206–0.826)	0.012	2.188 (0.884–5.418)	0.090

Use of mosquito repellent	Yes	1 (0.3)	19 (5.7)	Ref		Ref	
	No	41 (12.3)	271 (81.6)	0.348 (0.045–2.669)	0.310		

Use of IRS	Yes	13 (3.9)	125 (37.7)	Ref		Ref	
	No	29 (8.7)	165 (49.7)	0.592 (0.296–1.185)	0.139	1.285 (0.480–3.440)	0.617

*Note:* Ref = Reference category.

Abbreviations: AOR, adjusted odds ratio; CI, confidence interval; COR, crude odds ratio; IRS, insecticidal residual spray; ITN, insecticide-treated net.

^∗^Significant association.

**Table 7:** Association among malaria, STH, and their co-infection with respect to anemia severity among pregnant women attending ANC at MTUTH (*n* = 332).

Variables	Category	Anemia				Odds ratio (95% CI)	
		Non-anemic	Mild	Moderate	Severe	AOR	*p* value
Malaria parasite infection	Present	20 (6.0)	28 (8.4)	22 (6.6)	11 (3.3)	2.904 (1.630–5.174)	0.001^**∗**^
	Absent	101 (30.4)	90 (27.1)	59 (17.8)	1 (0.3)	Ref	

STH infection	Yes	25 (7.5)	51 (15.4)	28 (8.4)	8 (2.4)	4.118 (2.493–6.802)	0.001^**∗**^
	No	96 (28.9)	67 (20.2)	53 (16.0)	4 (1.2)	Ref	

Malaria–STH co-infection	Yes	10 (3.0)	11 (3.3)	12 (3.6)	9 (2.7)	0.462 (0.190–1.124)	0.089
	No	111 (33.4)	108 (32.5)	68 (20.5)	3 (0.9)	Ref	

*Note:* Ref = Reference category.

Abbreviations: AOR, adjusted odds ratio; CI, confidence interval; COR, crude odds ratio.

^∗^Significant association.

## Data Availability

The dataset for this study is available from the corresponding author upon reasonable request.

## Nomenclature

ACT: Artemisinin combination therapy
ANC: Antenatal clinic
AOR: Adjusted odds ratio
CI: Confidence interval
CHW: Community health workers
COR: Crude odds ratio
DHIS: Demographic health information system
Hb: Hemoglobin
HIV: Human immunodeficiency virus
IRS: Insecticidal residual spray
ITN: Insecticide-treated net
MTUTH: Mizan–Tepi University Teaching Hospital
NACOSTI: National Council of Science, Technology, and Innovation
NGO: Nongovernmental organizations
RBC: Red blood cells
SP: Sulfoxide pyrithiamine
SPSS: Statistical Package for Social Sciences
SSA: Sub-Sahara Africa
STH: Soil-transmitted helminth
TB: Tuberculosis
UNICEF: United Nations Children's Emergency Fund
UNDP: United Nation Development Program
USD: U.S. dollar
WHO: World Health Organization

## References

[1] Tadesse Boltena M., El-Khatib Z., Kebede A. S. (2022). Malaria and Helminthic Co-Infection During Pregnancy in Sub-Saharan Africa: A Systematic Review and Meta-Analysis. *International Journal of Environmental Research and Public Health*.

[2] Strunz E. C., Addiss D. G., Stocks M. E., Ogden S., Utzinger J., Freeman M. C. (2014). Water, Sanitation, Hygiene, and Soil-Transmitted Helminth Infection: A Systematic Review and Meta-Analysis. *PLoS Medicine*.

[3] World Health Organization (2013). Schistosomiasis: Progress Report 2001–2011, Strategic Plan 2012–2020.

[4] Das J. K., Lakhani S., Rahman A. R. (2024). Malaria in Pregnancy: Meta-Analyses of Prevalence and Associated Complications. *Epidemiology and Infection*.

[5] World Health Organization (Who) (2021). Global Prevalence of Anemia in Pregnant Women. *Global Health Observatory Data*.

[6] Stevens G. A., Finucane M. M., De-Regil L. M. (2013). Global, Regional, and National Trends in Haemoglobin Concentration and Prevalence of Total and Severe Anaemia in Children and Pregnant and Non-Pregnant Women for 1995–2011: A Systematic Analysis of Population-Representative Data. *Lancet Global Health*.

[7] Chrispinus Siteti M., Namasaka S. D., Ariya O. P., Injete S. D., Wanyonyi W. A. (2014). Anaemia in Pregnancy: Prevalence and Possible Risk Factors in Kakamega County, Kenya. *Science Journal of Public Health*.

[8] Nyambura A. W., Gicheru M. M., Kabiru E. W. (2019). Effects of HIV and Intestinal Parasites Co-Infection on Hematological Parameters Among Pregnant Women Attending Selected Health Facilities in Nyeri County, Kenya. *African Journal of Health Sciences*.

[9] Obeagu G. U., Obeagu E. I. (2025). Complications of Anemia in Pregnancy: An Updated Overview for Healthcare Professionals. *Medicine*.

[10] Stephen G., Mgongo M., Hussein Hashim T., Katanga J., Stray-Pedersen B., Msuya S. E. (2018). Anaemia in Pregnancy: Prevalence, Risk Factors, and Adverse Perinatal Outcomes in Northern Tanzania. *Anemia*.

[11] Greenwood B., Bojang K., Whi ty C. J. (2005). Targett GA Malaria. *The Lance*.

[12] Miller E. M. (2016). The Reproductive Ecology of Iron in Women. *American Journal of Physical Anthropology*.

[13] Okube O. T., Mirie W., Odhiambo E., Sabina W., Habtu M. (2016). Prevalence and Factors Associated With Anaemia Among Pregnant Women Attending Antenatal Clinic in the Second and Third Trimesters at Pumwani Maternity Hospital, Kenya. *Open Journal of Obstetrics and Gynecology*.

[14] Egwunyenga A. O., Ajayi J. A., Nmorsi O. P., Duhlinska-Popova D. D. (2001). Plasmodium/Intestinal Helminth Co-Infections Among Pregnant Nigerian Women. *Memorias do Instituto Oswaldo Cruz*.

[15] Mbule A. M., Byaruhanga Y. B., Kabahenda M., Lubowa A. (2013). Determinants of Anaemia Among Pregnant Women in Rural Uganda. *Rural and Remote Health*.

[16] Ojurongbe O., Okorie P. N., Opatokun R. L. (2018). Prevalence and Associated Factors of Plasmodium Falciparum and Soil Transmitted Helminth Infections Among Pregnant Women in Osun State, Nigeria. *African Health Sciences*.

[17] Osarfo J., Ampofo G. D., Tagbor H. (2022). Trends of Malaria Infection in Pregnancy in Ghana Over the Past Two Decades: A Review. *Malaria Journal*.

[18] Tamir Z., Animut A., Dugassa S. (2025). *Plasmodium* and Intestinal Parasite Infections Among Pregnant Women at First Antenatal Care Contact in Northwest Ethiopia: A Study of Prevalence and Associated Risk Factors. *PLoS One*.

[19] Erhabo O., Abdullahi A., Tosan E., Charles A. (2019). Risk Factors Associated With Malaria Infection Among Pregnant Women of African Descent in Specialist Hospital Sokoto, Nigeria. *Obstetrics & Gynecology International Journal*.

[20] Ogomaka I., Obeagu E. I. (2021). Malaria in Pregnancy Amidst Possession of Insecticide Treated Bed Nets (Itns) in Orlu LGA of Imo State, Nigeria. *Journal of Pharmaceutical Research International*.

[21] Demoze L., Adane K. C., Gizachew N., Tesfaye A. H., Yitageasu G. (2024). Utilization of Insecticide-Treated Nets Among Pregnant Women in East Africa: Evidence From a Systematic Review and Meta-Analysis. *BMC Public Health*.

[22] Agomo C. O., Oyibo W. A. (2013). Factors Associated with Risk of Malaria Infection Among Pregnant Women in Lagos, Nigeria. *Infectious Diseases of Poverty*.

[23] World Health Organization (2023). *Soil-Transmitted Helminth Infections*.

[24] World Health Organization (2019). *Water, Sanitation, Hygiene and Health: A Primer for Health Professionals*.

[25] Bangert M., Bancalari P., Mupfasoni D., Mikhailov A., Gabrielli A. F., Montresor A. (2019). Provision of Deworming Intervention to Pregnant Women by Antenatal Services in Countries Endemic for Sthiasis. *PLoS Neglected Tropical Diseases*.

[26] WHO (2023). WHO STH, CDC. Parasites-Soil-Transmitted Helminths.

[27] Caraballo H., King K. (2014). Emergency Department Management of Mosquito-Borne Illness: Malaria, Dengue, and West Nile Virus. *Emergency Medicine Practice*.

[28] WHO (2023). WHO Malaria, CDC. Malaria-Epidemiology and Global Burden.

[29] Getachew M., Tafess K., Zeynudin A., Yewhalaw D. (2013). Prevalence STHiasis and Malaria co-infection Among Pregnant Women and Risk Factors in Gilgel Gibe Dam Area, Southwest Ethiopia. *BMC Research Notes*.

[30] Naing L., Nordin R. B., Abdul Rahman H., Naing Y. T. (2022). Sample Size Calculation for Prevalence Studies Using Scalex and ScalaR Calculators. *BMC Medical Research Methodology*.

[31] Tan J., He G., Qi Y. (2020). Prevalence of Anemia and Iron Deficiency Anemia in Chinese Pregnant Women (IRON WOMEN): A National Cross-Sectional Survey. *BMC Pregnancy and Childbirth*.

[32] Karami M., Chaleshgar M., Salari N., Akbari H., Mohammadi M. (2022). Global Prevalence of Anemia in Pregnant Women: A Comprehensive Systematic Review and Meta-Analysis. *Maternal and Child Health Journal*.

[33] Mostafa E., Mohammed H. F., Mohammed E. M., Mohamed Ali A. S. (2022). Prevalence and Risk Factors of Iron Deficiency Anaemia With Pregnancy at Minia University Hospital. *Minia Journal of Medical Research*.

[34] Tesfaye T. S., Tessema F., Jarso H. (2020). Prevalence of Anemia and Associated Factors Among “Apparently Healthy” Urban and Rural Residents in Ethiopia: A Comparative Cross-Sectional Study. *Journal of Blood Medicine*.

[35] Sabina Azhar B., Islam M. S., Karim M. R. (2021). Prevalence of Anemia and Associated Risk Factors Among Pregnant Women Attending Antenatal Care in Bangladesh: A Cross-Sectional Study. *Primary Health Care Research & Development*.

[36] Tay S. C. K., Nani E. A., Walana W. (2017). Parasitic Infections and Maternal Anaemia Among Expectant Mothers in the Dangme East District of Ghana. *BMC Research Notes*.

[37] Zegeye A. F., Wassie M., Tamir T. T. (2025). Malaria-Anemia Comorbidity and Its Determinants Among Pregnant Women in High-and Moderate-Malaria-Risk Countries in Sub-Saharan Africa. *Infectious Diseases of Poverty*.

[38] Iwunze J., Amaechi A., Nwoke M., Njoku F., Okeke O. (2019). Prevalence of Malaria Among Users and Non Users of ITNs in Obowo Local Government Area Imo State, Nigeria. *J Entomol Zool Stud.*.

[39] Wanyonyi W. A., Mulambalah C. S., Mulama D. H., Omukunda E., Siteti D. I. (2018). Malaria and Geohelminthiasis Coinfections in Expectant Women: Effect on Maternal Health and Birth Outcomes in a Malaria Endemic Region in Kenya. *Journal of parasitology research*.

[40] Agboli E., Tay S., Obirikorang C., Aidoo E. (2016). Malaria and Intestinal Parasites in Pregnant and Non-Pregnant Women: A Comparative Study at the University Hospital, Kumasi, Ghana. *Journal of Medicine and Biomedical Sciences*.

[41] Geleta G., Ketema T. (2017). Prevalence of Malaria and Frequency of Severe Symptoms Among Pregnant Women in Pawe Hospital, North Western Ethiopia. *Ann Clin Pathol*.

[42] Kirui E. (2024). Determination of the Prevalence and Associated Factors of Malaria Among Pregnant Women in Kenya.

[43] World Health Organization (2014). World Malaria Report.

[44] Anchang-Kimbi J. K., Elad D. M., Sotoing G. T., Achidi E. A. (2017). Co-Infection With *Schistosoma haematobium* and *Plasmodium falciparum* and Anaemia Severity Among Pregnant Women in Munyenge, Mount Cameroon Area: A Cross‐Sectional Study. *Journal of Parasitology Research*.

[45] Ndegwa S. K. (2019). Anemia & Its Associated Factors Among Pregnant Women Attending Antenatal Clinic at Mbagathi County Hospital, Nairobi County, Kenya. *African Journal of Health Sciences*.

[46] Gebrehiwet M. G., Medhaniye A. A., Alema H. B. (2019). Prevalence and Associated Factors of Soil Transmitted Helminthes Among Pregnant Women Attending Antenatal Care in Maytsebri Primary Hospital, North Ethiopia. *BMC Research Notes*.

[47] Derso A., Nibret E., Munshea A. (2016). Prevalence of Intestinal Parasitic Infections and Associated Risk Factors Among Pregnant Women Attending Antenatal Care Center at Felege Hiwot Referral Hospital, Northwest Ethiopia. *BMC Infectious Diseases*.

[48] Njeru A., Mutuku F., Muriu S. (2019). Status of STH Among Pregnant Women Attending Antenatal Clinic in Kilifi County Hospital, Kenya. *bioRxiv*.

[49] Wekesa A., Mulambalah C., Muleke C., Odhiambo R. (2014). Intestinal Helminth Infections in Pregnant Women Attending Antenatal Clinic at Kitale District Hospital, Kenya. *Journal of parasitology research*.

[50] Salami J. S., Ojoko A., Isyaku A., Odewole C., Ogwu M. (2025). Prevalence of Malaria and Intestinal Helminths Co-Infection in Pregnant Women. *Kontagora Journal of Intellectual Discourse*.

[51] Gemechu T., Aliyo A., Husen O., Jarso H., Assefa L. (2024). Co-Infection of Plasmodium and Soil-Transmitted Helminth Among Pregnant Women in Abaya District, South Ethiopia: A Community-Based Study. *IJID Regions*.

[52] Yatich N. J., Yi J., Agbenyega T. (2009). Malaria and Intestinal Helminth co-Infection Among Pregnant Women in Ghana: Prevalence and Risk Factors. *The American Journal of Tropical Medicine and Hygiene*.

[53] Chessed G., Ibeh G., Daskum A., Manu J. (2021). Malaria and Intestinal Helminth Co-Infection Among Pregnant Women Attending Federal Teaching Hospital Gombe, Gombe State, Nigeria. *Nigerian Journal of Parasitology*.

[54] Degarege A., Erko B. (2016). Epidemiology of *Plasmodium* and Helminth Co-Infection and Possible Reasons for Heterogeneity. *BioMed Research International*.

[55] Fernández-Niño J. A., Idrovo A. J., Cucunubá Z. M. (2012). Paradoxical Associations Between STHsand *Plasmodium falciparum* Infection. *Transactions of the Royal Society of Tropical Medicine and Hygiene*.

[56] Tegegne Y., Asmelash D., Ambachew S., Eshetie S., Addisu A., Jejaw Zeleke A. (2019). The Prevalence of Malaria Among Pregnant Women in Ethiopia: A Systematic Review and Meta‐Analysis. *Journal of parasitology research*.

[57] McClure E. M., Meshnick S. R., Mungai P. (2014). The Association of Parasitic Infections in Pregnancy and Maternal and Fetal Anemia: A Cohort Study in Coastal Kenya. *PLoS Neglected Tropical Diseases*.

